# The complete mt genomes of *Lutzia halifaxia*, *Lt. fuscanus* and *Culex pallidothorax* (Diptera: Culicidae) and comparative analysis of 16 *Culex* and *Lutzia* mt genome sequences

**DOI:** 10.1186/s13071-019-3625-2

**Published:** 2019-07-26

**Authors:** Ling Sun, Ting-Jing Li, Wen-Bo Fu, Zhen-Tian Yan, Feng-Ling Si, Yu-Juan Zhang, Qi-Meng Mao, Bruna Demari-Silva, Bin Chen

**Affiliations:** 1 Chongqing Key Laboratory of Vector Insects, Institute of Entomology and Molecular Biology, College of Life Sciences, Chongqing Normal University, Chongqing, 401331 P. R. China; 2 Faculdade de Saúde Pública, Departamento de Epidemiologia, Universidade de São Paulo, Avenida Dr. Arnaldo, 715, São Paulo, Brazil

**Keywords:** *Lutzia halifaxia*, *Lutzia fuscanus*, *Culex pallidothorax*, *Lutzia*, *Culiciomyia*, *Culex*, Mitochondrial genome, Phylogeny

## Abstract

**Background:**

Despite the medical importance of the genus *Culex*, the mitochondrial genome (mt genome) characteristics of *Culex* spp. are not well understood. The phylogeny of the genus and particularly the generic status of the genus *Lutzia* and the subgenus *Culiciomyia* remain unclear.

**Methods:**

The present study sequenced and analyzed the complete mt genomes of *Lutzia halifaxia*, *Lutzia fuscanus* and *Cx.* (*Culiciomyia*) *pallidothorax* and assessed the general characteristics and phylogenetics of all known 16 mt genome sequences for species in the genera *Culex* and *Lutzia.*

**Results:**

The complete mt genomes of *Lt. halifaxia*, *Lt. fuscanus* and *Cx. pallidothorax* are 15,744, 15,803 and 15,578 bp long, respectively, including 13 PCGs, 22 tRNAs, two tRNAs and a control region (CR). Length variations in the *Culex* and *Lutzia* mt genomes involved mainly the CR, and gene arrangements are the same as in other mosquitoes. We identified four types of repeat units in the CR sequences, and the poly-T stretch exists in all of these mt genomes. The repeat units of CR are conserved to different extent and provide information on their evolution. Phylogenetic analyses demonstrated that the Coronator and Sitiens groups are each monophyletic, whereas the monophyletic status of the Pipiens Group was not supported; *Cx. pallidothorax* is more closely related to the Sitiens and Pipiens groups; and both phylogenetics analysis and repeat unit features in CR show that *Lutzia* is a characteristic monophyletic entity, which should be an independent genus.

**Conclusions:**

To our knowledge, this is the first comprehensive review of the mt genome sequences and taxonomic discussion based on the mt genomes of *Culex* spp. and *Lutzia* spp. The research provides general information on the mt genome of these two genera, and the phylogenetic and taxonomic status of *Lutzia* and *Culiciomyia*.

**Electronic supplementary material:**

The online version of this article (10.1186/s13071-019-3625-2) contains supplementary material, which is available to authorized users.

## Background

The genus *Culex* is the largest genus in the Culicidae in terms of the number of species and is distributed worldwide [1]. Some *Culex* species are important vectors of infectious and arboviral diseases such as epidemic encephalitis and lymphatic filariasis [1]. The genus *Lutzia* was established by Theobald in 1903 [2] and was then reduced to a subgenus of the genus *Culex* by Edwards in 1932 [3]. Subsequent authors treated it as a subgenus until 2003, when Tanaka formally restored *Lutzia* to its original generic status [4]. However, the taxonomic level of *Lutzia* has remained controversial. For example, phylogenetic analysis based on larval and adult morphological characteristics placed *Lutzia* outside the clade that comprised the genus *Culex* (including representative species in all 26 subgenera) [5, 6]. In contrast, molecular phylogenetic analyses placed *Lutzia* among species of the genus *Culex* based on ITS1 and ITS2 sequences and *cox*1 [7–9]. The molecular phylogenetic analysis based on ITS1 and ITS2 sequences using neighbor-joining approach indicated that the genus *Lutzia* (one species included) formed the sister group to the subgenus *Culex* (11 species included) [7]. In contrast, the analysis based on 478 bp of *cox*1 using Bayesian method suggested that the genus *Culex* (17 species included) is paraphyletic relative to *Lutzia* (one species included) [8]. The analysis based on ITS2 using neighbor-joining approach showed that the genus *Lutzia* (one species included) was placed inside the genus *Culex* (16 species included) [9].

*Lutzia* is distributed in the Afrotropical, Oriental, southern Palaearctic, Australasian and Neotropical regions and has eight known species, with only two species (*Lutzia halifaxia* and *Lutzia fuscanus*) recorded in China. Subgenus *Culiciomyia* was established by Edwards in 1921 [10] and has 55 known species with a geographical distribution in Afrotropical, Oriental and Australasian regions [1]. *Culex pallidothorax* in the subgenus *Culiciomyia* was grouped into the subgenus *Culex* with a low bootstrap support of 11% based on the results of phylogenetic analysis of *cox*1 sequences [11]. The mitochondrial genome (mt genome) sequence of the subgenus has not yet been investigated.

Mitochondria are related to various biological processes, from power production to programmed cell death and ageing [12]. Mitochondrial DNA (mtDNA) sequences have been widely used as molecular markers for the identification of organisms and in research investigations on insect population genetics and phylogenetics [13–19]. As of 20 March 2018, a total of 13 different mt genome sequences have been reported in the genus *Culex*, and these sequences are all from nine species/subspecies within the subgenus *Culex* (*Cx. camposi*, *Cx. coronator*, *Cx. gelidus*, *Cx*. *pipiens pallens*, *Cx*. *pipiens pipiens*, *Cx*. *quinquefasciatus*, *Cx*. *tritaeniorhynchus*, *Cx. usquatus* and *Cx. usquatissimus*) [20–23]. To date, no mt genome sequence has been reported for *Lutzia* or *Culiciomyia*.

In the present study, we sequenced and analyzed the complete mt genomes of *Lt. halifaxia* and *Lt. fuscanus* in *Lutzia* and *Culex* (*Culiciomyia*) *pallidothorax* in the subgenus *Culiciomyia*, comprehensively analyzed the characteristics of all 16 mt genome sequences in the genus *Culex* available to date (including three mt genome sequences obtained in the present study), and conducted phylogenetic reconstruction using these 16 mt genomes. The study also generated insights into the taxonomic status and position of *Lutzia* and *Culiciomyia*.

## Methods

### Sample collection and total DNA extraction

Specimens of *Lt. halifaxia* and *Cx.* (*Culiciomyia*) *pallidothorax* were collected from Leishan County, Guizhou Province, China (26°29′27″N, 108°09′27″E) in July 2015. Specimens of *Lt. fuscanus* specimens were collected from Shuicheng County, Guizhou Province, China (26°35′40″N, 104°48′07″E) in August 2015. All collected samples were stored in 100% alcohol and stored at − 20 °C until use. These three species of mosquitoes were initially identified using morphological characteristics [24] and then confirmed by sequencing the *cox*1 and ITS2 loci as reported elsewhere [25]. Total DNA was separately extracted from a female adult of each species using a TIANamp Genomic DNA Kit (TianGen, Shanghai, China) following the manufacturerʼs instructions, and then total DNAs were preserved at − 80 °C for subsequent mt genome sequencing.

### Mt genome sequencing, assembly and annotation

The mt genome fragments of these three species were amplified by the universal primers for Diptera [26]. Due to the amplification difficulty of the control region (CR) of *Lt. halifaxia* and *Lt. fuscanus* mt genomes, one additional pair of primers (F: 5′-TCA ATT TAC TAT TAT ATT TAT TGG AG-3′ and R: 5′-TAA TTT CAA TAG TTT GTC CAT GTA-3′) was designed with online Primer3 (http://biotools.umassmed.edu/bioapps/primer3_www.cgi) according to known Culicidae mt genomes and applied to fill the sequence gap of the CR. All PCR amplifications were performed in 25 μl reactions containing 4 μl of dNTPs, 1 μl of each primer, 2.5 μl of 10× LA PCR buffer I, 1–2 μl of DNA template, 0.25 μl of LA Taq polymerase (TaKaRa, Dalian, China) and 14.25–15.25 μl ddH_2_O. The PCR amplification conditions were as follows: an initial denaturation at 94 °C for 1 min; 35 cycles of 94 °C for 40 s (denaturation), 47–58 °C for 45 s (annealing) and 68 °C for 1 min (extension); followed by a final extension at 72 °C for 10 min. All PCR fragments were successfully amplified using the extracted DNA template, but the CR was cloned into the vector pMD-19T (TaKaRa) and then amplified due to extensive sequence variations. All PCR fragments were subsequently purified with a QIAquick PCR Purification Kit (Qiagen, Hilden, Germany) and were sequenced using a DNA Sequencer (ABI3730) at Life Technologies™ Company (Shanghai, China) in both directions.

The obtained sequences were assembled using DNAMANx software. All genes [13 protein-coding genes (PCGs) and two ribosomal RNA genes (rRNAs)] and the CR were identified by comparing with the corresponding sequences in other known *Culex* mt genomes with ClustalX [27], whereas transfer RNA genes (tRNAs) were identified using tRNAscan-SE Search Server v.1.21 (http://lowelab.ucsc.edu/tRNAscan-SE/) [28]. Some tRNAs that could not be identified by tRNAscan-SE were diagnosed by the multiple sequence alignment with the tRNA sequences of known *Culex* mt genomes. The base composition, relative synonymous codon usage (RSCU), and amino acid content were computed with MEGA v.5.0 software [29]. AT-skew [(A − T)/(A + T)] and GC-skew [(G − C)/(G + C)] were estimated in order to investigate nucleotide composition bias [30]. The graphical maps of the mt genomes were visualized with the CGView Comparison Tool [31]. The three-dimensional scatter plot of the AT-skew and GC-skew of these 16 mt genomes was drawn using Origin Pro v.9.0 [32]. The tandem repeats in the CRs were identified using the Tandem Repeats Finder program [33]. The secondary structures of tRNAs were predicted by tRNAscan-SE Search Server v.1.21.

### Phylogenetic analysis

Phylogenetic analysis of the 16 *Culex* mt genomes (including three mt genomes produced in the present study and 13 *Culex* mt genomes deposited in GenBank; accession numbers are listed in Table 1) were performed using the Bayesian Inference (BI) analysis in MrBayes v.3.2.6 [34]. The amino acid sequence of each protein-coding gene was aligned individually based on codon-based multiple alignments using the MAFFT algorithm within the TranslatorX server (www.translatorx.co.uk) [35]. Poorly aligned sites were removed from the amino acid alignment before translating back to nucleotides using GBlocks in TranslatorX with default settings. The nucleotide sequences of the 13 PCGs were applied in the analysis because these are considered most suitable for inferring the phylogenetic relationships of known mt genome sequences of genus *Culex* [22]. The mt genome sequences of *Anopheles gambiae* (GenBank: NC002084) and *Aedes aegypti* (GenBank: NC010241) were used as outgroups. The best-fit model for each gene was chosen under the Akaike information criterion by Modeltest [36]. The concatenated matrix of the 13 PCGs was used to carry out the BI analysis. For the latter, two independent runs were performed, each with three hot chains and one cold chain, with posterior distributions estimated using Markov Chain Monte Carlo (MCMC) sampling. The MCMC chains were set for 5,000,000 generations, with tree sampling every 1000 steps and a relative ‛burn-inʼ of 25%. The convergence of the two runs was evaluated by average standard deviation of split frequencies (< 0.01). The phylogenetic tree was drawn in FigTree v.1.4.2 (http://tree.bio.ed.ac.uk/software/figtree/).

**Table 1:** Detailed sequence information of 16 mt genomes of species in genera *Culex* and *Lutzia*

Genus/Subgenus	Species	Total size (bp)	PCGs size (bp)	tRNA size (bp)	rRNA size (bp)	CR size (bp)	GenBank ID	Reference
*Lutzia*	*Lt. halifaxia*	15,744	11,226	1484	2134	899	MH316119	This study
	*Lt. fuscanus*	15,803	11,218	1481	2126	920	MH316118	This study
*Culiciomyia*	*Cx. pallidothorax*	15,578	11,222	1482	2128	724	KY400104	This study
*Culex*	*Cx. camposi*	15,570	11,228	1483	2124	719	NC_036008.1	[20]
	*Cx. coronator*	15,576	11,228	1482	2124	725	NC_036006.1	[20]
	*Cx. gelidus*	15,600	11,230	1414	2143	721	KX753344	[22]
	*Cx. pipiens pallens*	15,617	11,234	1482	2138	747	KT851543.1	[21]
	*Cx. pipiens pipiens*	14,856	11,188	1475	2118	^a^	NC_015079.1	GenBank^b^
	*Cx. pipiens* TU	14,856	11,216	1475	2118	^a^	HQ724616.1	GenBank^b^
	*Cx. quinquefasciatus*	15,587	11,220	1467	2137	704	NC_014574.1	[19]
	*Cx. quinquefasciatus* USA	14,856	11,216	1476	2118	^a^	HQ724617.1	GenBank^b^
	*Cx. tritaeniorhynchus* CQ	14,844	11,219	1498	2143	^a^	KT851544.1	[21]
	*Cx. tritaeniorhynchus* JS	14,861	11,222	1473	2128	^a^	NC_028616.1	GenBank^b^
	*Cx. usquatissimus* AC	15,573	11,228	1482	2124	721	MF040165.1	[20]
	*Cx. usquatissimus* RO	15,574	11,228	1483	2124	722	NC_036007.1	[20]
	*Cx. usquatus*	15,573	11,228	1483	2124	719	NC_036005.1	[20]

^a^Does not harbor the CR

^b^Reported only in GenBank

## Results

### Genome organization and nucleotide composition

**Fig. 1:**
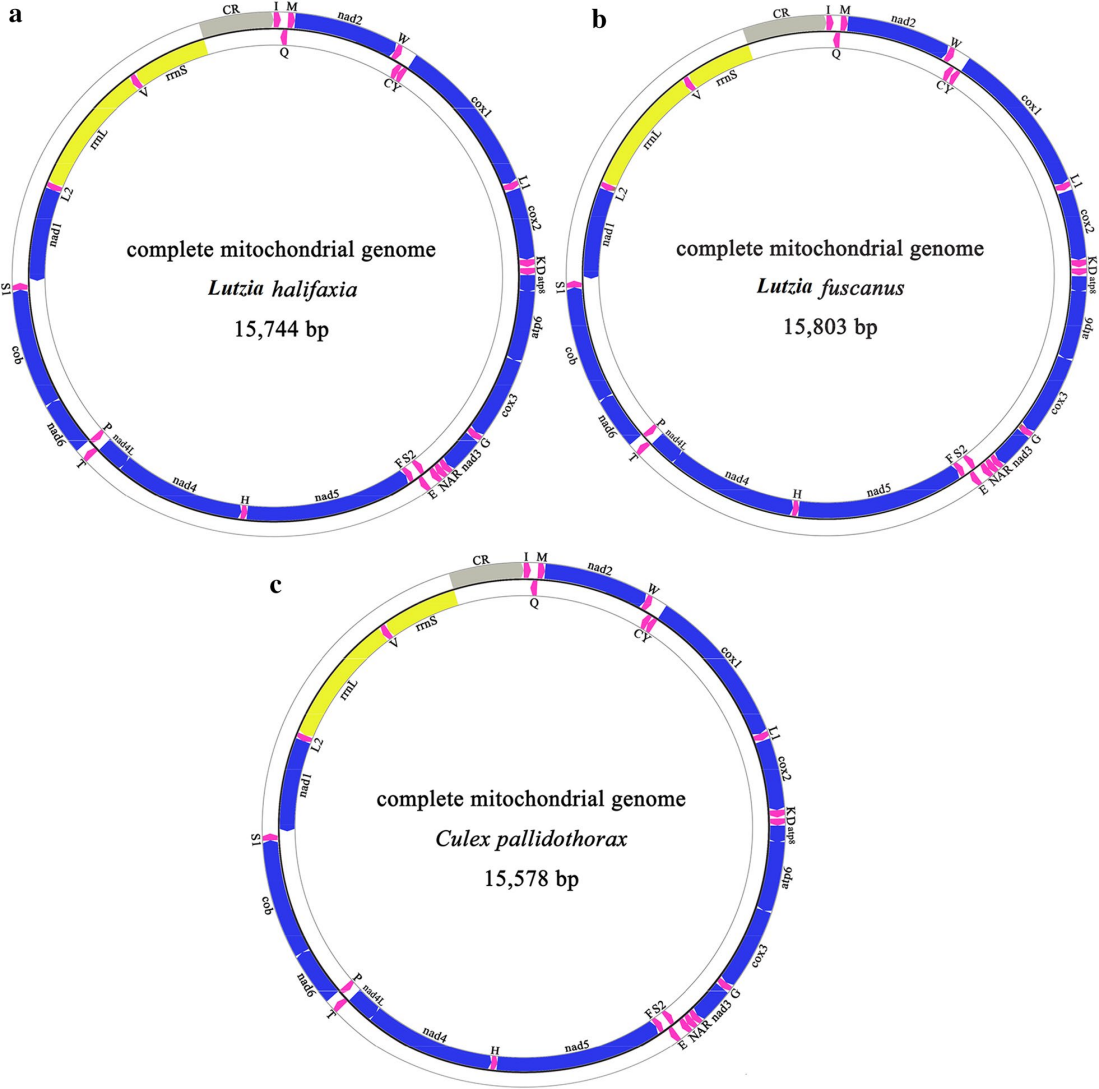
Mt genome structure of *Lt. halifaxia* (**a**), *Lt. fuscanus* (**b**) and *Cx. pallidothorax* (**c**). The blue-, pink-, yellow- and gray-filled blocks indicate PCGs, tRNAs, rRNAs and CR, respectively. The genes on the outer circle are located on the J-strand, whereas the genes on the inner circle are located on the N-strand. L, L2, S1 and S2 represent the tRNAs *trnL*1, *trnL*2, *trnS*1 and *trnS*2, respectively. Arrows indicate the transcriptional direction of mitochondrial genes

The complete length of the mt genomes of *Lt. halifaxia* (GenBank: MH316119), *Lt. fuscanus* (GenBank: MH316118) and *Cx. pallidothorax* (GenBank: KY400104) was 15,744, 15,803 and 15,578 bp, respectively (Fig. 1). All mt genomes included 37 genes (13 PCGs, 22 tRNAs and 2 rRNAs) and a control region (CR), with 9 PCGs and 13 tRNAs encoded on the majority strand (J-strand) and 4 PCGs, 9 tRNAs and 2 rRNAs on the minority strand (N-strand). Comparison of the mt genomes of the two *Lutzia* spp. with nine *Culex* spp. which all have complete mt genome sequences indicated that those of *Lt. halifaxia* and *Lt. fuscanus* are 127–233 bp longer (Table 1). The PCGs, tRNAs and rRNAs are conservative in length, and the CRs are relatively variable in length, with the *Lt. halifaxia* and *Lt. fuscanus* CRs being much longer (898 and 920 bp, respectively) than the nine *Culex* mt genome CRs, which ranged from 704 bp in *Cx. quinquefasciatus* to 747 bp in *Cx. p. pallens* (Additional file 1: Table S1). Similarly to the 13 published *Culex* mt genomes, the nucleotide compositions of *Lt. halifaxia*, *Lt. fuscanus* and *Cx. pallidothorax* mt genomes are biased toward A and T, with A being the most favored nucleotide and C as the least favored. The observed adenine + thymine (AT) content of the mt genomes was high, accounting for 77.96% (A = 39.28%; T = 38.68%; G = 9.30%; C = 12.74%), 78.40% (A = 39.70%; T = 38.70%; G = 9.10%; C = 12.60%) and 78.50% (A = 39.70%; T = 38.80%; G = 9.10%; C = 12.70%) in *Lt. halifaxia*, *Lt. fuscanus* and *Cx. pallidothorax*, respectively (Additional file 1: Table S1).

**Fig. 2:**
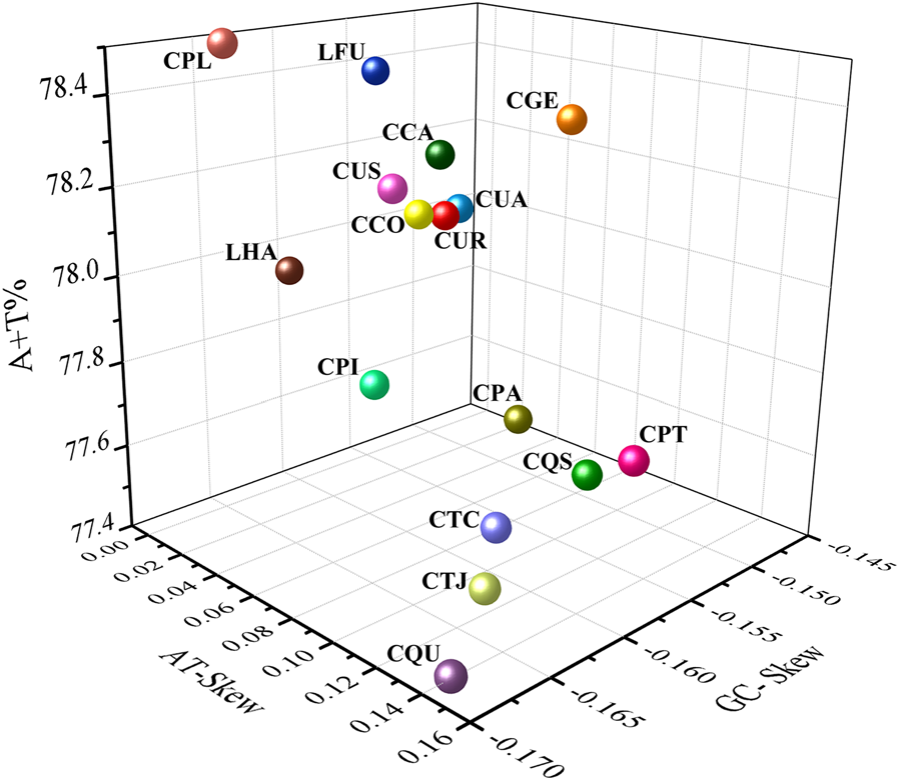
Three-dimensional scatterplot of the AT-Skew, GC-Skew and AT% of 16 *Culex* and *Lutzia* mt genome sequences. *Abbreviations*: CCA, *Cx. camposi*; CCO, *Cx. coronator*; LFU, *Lt. fuscanus*; CGE, *Cx. gelidus*; LHA, *Lt. halifaxia*; CPA, *Cx. p. pallens*; CPI, *Cx. p. pipiens*; CPL, *Cx. pallidothorax*; CPT, *Cx. pipiens* TU; CQS, *Cx. quinquefasciatus* USA; CQU, *Cx. quinquefasciatus*; CTC, *Cx. tritaeniorhynchus* CQ; CTJ, *Cx. tritaeniorhynchus* JS; CUA, *Cx. usquatissimus* AC; CUR, *Cx. usquatissimus* RO; CUS, *Cx. usquatus*

The three-dimensional scatter plot of the AT content, AT-skew and GC-skew in the 14 *Culex* spp. and 2 *Lutzia* spp. mt genomes is shown in Fig. 2. The AT-skew of *Lt. halifaxia* (0.0078) and *Cx. pallidothorax* mt genome (0.0078) are lower than the average AT-skew of all investigated mt genomes (0.0107), whereas the AT-skew of *Lt. fuscanus* mt genome (0.0128) is higher than the average AT-skew value. The GC-skew in *Lt. halifaxia* (-0.1613) and *Cx. pallidothorax* (-0.1651) are a bit lower than the average investigated GC-skew value (-0.1572), whereas the GC-skew of *Lt. fuscanus* mt genome (-0.1559) is slightly higher than the average GC-skew value. In general, the AT-skew and GC-skew are highly variable in the investigated mt genomes. For example, species of the Coronator group [*Cx. camposi*, *Cx. coronator*, *Cx. usquatissimus* AC (geographical name as published), *Cx. usquatissimus* RO and *Cx. usquatus*)] have similar AT content and AT/GC-skew, which are closely distributed in the three-dimensional scatter plot, whereas the species of the Pipiens group (*Cx*. *p*. *pallens*, *Cx*. *p*. *pipiens*, *Cx*. *pipiens* TU, *Cx. quinquefasciatus* and *Cx. quinquefasciatus* USA) are widely distributed in the plot for AT content, AT-skew and GC-skew (Fig. 2).

### Protein-coding genes

The total nucleotide length of the 13 PCGs of *Lt. halifaxia*, *Lt. fuscanus* and *Cx. pallidothorax* was 11,226, 11,218 and 11,222 bp, respectively, falling within the range of total nucleotide length variations of the 13 PCGs in the 16 *Culex* spp. mt genomes (from 11,188 bp in *Cx. p. pipiens* to 11,234 bp in *Cx. p. pallens*) (Table 1). ATN is used as the start codon of *Lt. halifaxia*, *Lt. fuscanus* and *Cx. pallidothorax* PCGs, except for *cox*1, which uses TCG as a start codon (Tables 2, 3, 4). Specifically, six PCGs (*cox*2, *cox*3, *atp*6, *nad*4, *nad*4*L* and *cob*) begin with ATG, four PCGs (*atp*8, *nad*1, *nad*3 and *nad*6) with ATA and two PCGs (*nad*2 and *nad*5) with ATC. The most frequently used codon among the PCGs is TAA, followed by TA and T. Among the 16 investigated mt genomes, ATN is the most frequently used start codon, followed by TCG, and TAA is the most frequently used stop codon, followed by TA and T.

**Table 2:** Organization of the *Lt. halifaxia* mt genome

Gene	Strand	Position	Size (bp)	Space (+)/Overlap (−)	Anticodon	Codon	
						Start	Stop
*trnI*	J	1–69	69		GAT		
*trnQ*	N	70–139	70	0	TTG		
*trnM*	J	150–218	69	10	CAT		
*nad*2	J	219–1241	1023	0		ATC	TAA
*trnW*	J	1242–1309	68	0	TCA		
*trnC*	N	1309–1374	66	− 1	GCA		
*trnY*	N	1375–1440	66	0	GTA		
*cox*1	J	1439–2975	1537	− 2		TCG	T
*trnL*2	J	2969–3034	66	− 7	TAA		
*cox*2	J	3042–3726	685	7		ATG	T
*trnK*	J	3727–3797	71	0	CTT		
*trnD*	J	3798–3865	68	0	GTC		
*atp*8	J	3 875–4027	153	9		ATA	TAA
*atp*6	J	4021–4701	681	− 7		ATG	TAA
*cox*3	J	4701–5489	789	− 1		ATG	TAA
*trnG*	J	5489–5555	67	− 1	TCC		
*nad*3	J	5553–5909	357	− 3		ATA	TAA
*trnA*	J	5908–5971	64	− 2	TCG		
*trnR*	J	5972–6037	66	0	TGC		
*trnN*	J	6038–6 104	67	0	GTT		
*trnS*1	N	6107–6173	67	2	GCT		
*trnE*	J	6175–6240	66	1	TTC		
*trnF*	N	6239–6305	67	− 2	GAA		
*nad*5	N	6280–8025	1746	− 26		ATC	TAA
*trnH*	N	8023–8090	68	− 3	GTG		
*nad*4	N	8090–9433	1344	− 1		ATG	TAA
*nad*4*L*	N	9427–9723	297	− 7		ATG	TAA
*trnT*	J	9729–9794	66	5	TGT		
*trnP*	N	9795–9860	66	0	TGG		
*nad*6	J	9866–10,384	519	5		ATA	TA
*cob*	J	10,400–11,536	1137	15		ATG	TAA
*trnS*2	J	11,536–11,601	66	− 1	TGA		
*nad*1	N	11,620–12,576	957	18		ATA	TAA
*trnL*1	N	12,571–12,638	68	− 6	TAG		
*rrnL*	N	12,639–13,975	1337	0			
*trnV*	N	13,976–14,047	72	0	TAC		
*rrnS*	N	14,048–14,844	797	0			
CR		14,845–15,744	899	0

**Table 3:** Organization of the *Lt. fuscanus* mt genome

Gene	Strand	Position	Size (bp)	Space (+)/Overlap (−)	Anticodon	Codon	
						Start	Stop
*trnI*	J	1–69	69		GAT		
*trnQ*	N	70–139	70	0	TTG		
*trnM*	J	150–218	69	10	CAT		
*nad*2	J	219–1241	1023	0		ATC	TAA
*trnW*	J	1242–1310	69	0	TCA		
*trnC*	N	1311–1376	66	0	GCA		
*trnY*	N	1377–1442	67	0	GTA		
*cox*1	J	1441–2977	1537	− 2		TCG	T
*trnL*2	J	2978–3044	67	0	TAA		
*cox*2	J	3053–3737	685	8		ATG	T
*trnK*	J	3738–3808	71	0	CTT		
*trnD*	J	3817–3883	67	8	GTC		
*atp*8	J	3893–4045	153	9		ATA	TAA
*atp*6	J	4039–4719	681	− 7		ATG	TAA
*cox*3	J	4719–5507	789	− 1		ATG	TAA
*trnG*	J	5507–5573	67	− 1	TCC		
*nad*3	J	5571–5927	357	− 3		ATA	TAA
*trnA*	J	5926–5989	64	− 2	TCG		
*trnR*	J	5990–6055	66	0	TGC		
*trnN*	J	6056–6122	67	0	GTT		
*trnS*1	N	6125–6191	67	2	GCT		
*trnE*	J	6193–6258	66	1	TTC		
*trnF*	N	6257–6323	67	− 2	GAA		
*nad*5	N	6324–8068	1745	0		ATC	TAA
*trnH*	N	8066–8131	66	− 3	GTG		
*nad*4	N	8131–9471	1344	− 1		ATG	TAA
*nad*4*L*	N	9468–9764	297	− 4		ATG	TAA
*trnT*	J	9770–9834	65	5	TGT		
*trnP*	N	9860–9925	66	25	TGG		
*nad*6	J	9931–10,446	515	5		ATA	TAA
*cob*	J	10,446–11,582	1137	− 1		ATG	TAA
*trnS*2	J	11,582–11,647	65	− 1	TGA		
*nad*1	N	11,666–12,621	957	18		ATA	TAA
*trnL*1	N	12,617–12,683	67	− 6	TAG		
*rrnL*	N	12,687–14,019	1333	3			
*trnV*	N	14,021–14,092	72	1	TAC		
*rrnS*	N	14,093–14,885	793	0			
CR		14,886–15,803	920	0

**Table 4:** Organization of the *Cx. pallidothorax* mt genome

Gene	Strand	Position	Size (bp)	Space (+)/Overlap (−)	Anticodon	Codon	
						Start	Stop
*trnI*	J	1–69	69		GAT		
*trnQ*	N	67–135	69	− 3	TTG		
*trnM*	J	140–208	69	4	CAT		
*nad*2	J	209–1230	1022	0		ATC	TA
*trnW*	J	1233–1301	69	2	TCA		
*trnC*	N	1302–1367	66	0	GCA		
*trnY*	N	1367–1432	66	− 1	GTA		
*cox*1	J	1431–2967	1537	− 2		TCG	T
*trnL*2	J	2968–3032	65	0	TAA		
*cox*2	J	3040–3724	685	7		ATG	T
*trnK*	J	3725–3795	71	0	CTT		
*trnD*	J	3806–3873	68	10	GTC		
*atp*8	J	3883**–**4036	154	9		ATA	TAA
*atp*6	J	4029–4709	681	− 8		ATG	TAA
*cox*3	J	4709–5 498	790	− 1		ATG	TAA
*trnG*	J	5497–5563	67	− 2	TCC		
*nad*3	J	5561–5917	357	− 3		ATA	TAA
*trnA*	J	5916–5979	64	− 2	TCG		
*trnR*	J	5980–6045	66	0	TGC		
*trnN*	J	6046–6112	67	0	GTT		
*trnS*1	N	6129–6196	68	18	GCT		
*trnE*	J	6184–6251	68	− 13	TTC		
*trnF*	N	6250–6316	67	− 2	GAA		
*nad*5	N	6317–8062	1746	0		ATC	TAA
*trnH*	N	8060–8125	66	− 3	GTG		
*nad*4	N	8125–9469	1345	− 1		ATG	TAA
*nad*4*L*	N	9463–9759	297	− 7		ATG	TAA
*trnT*	J	9765–9830	66	5	TGT		
*trnP*	N	9831–9896	66	0	TGG		
*nad*6	J	9902–10,417	516	5		ATA	TAA
*cob*	J	10,417–11,551	1135	− 1		ATG	TA
*trnS*2	J	11,552–11,617	66	0	TGA		
*nad*1	N	11,635–12,591	957	17		ATA	TAA
*trnL*1	N	12,586–12,652	67	− 6	TAG		
*rrnL*	N	12,654–13,989	1335	1			
*trnV*	N	13,990–14,061	72	0	TAC		
*rrnS*	N	14,062–14,854	793	0			
CR		14,855–15,578	724	0			

**Fig. 3:**
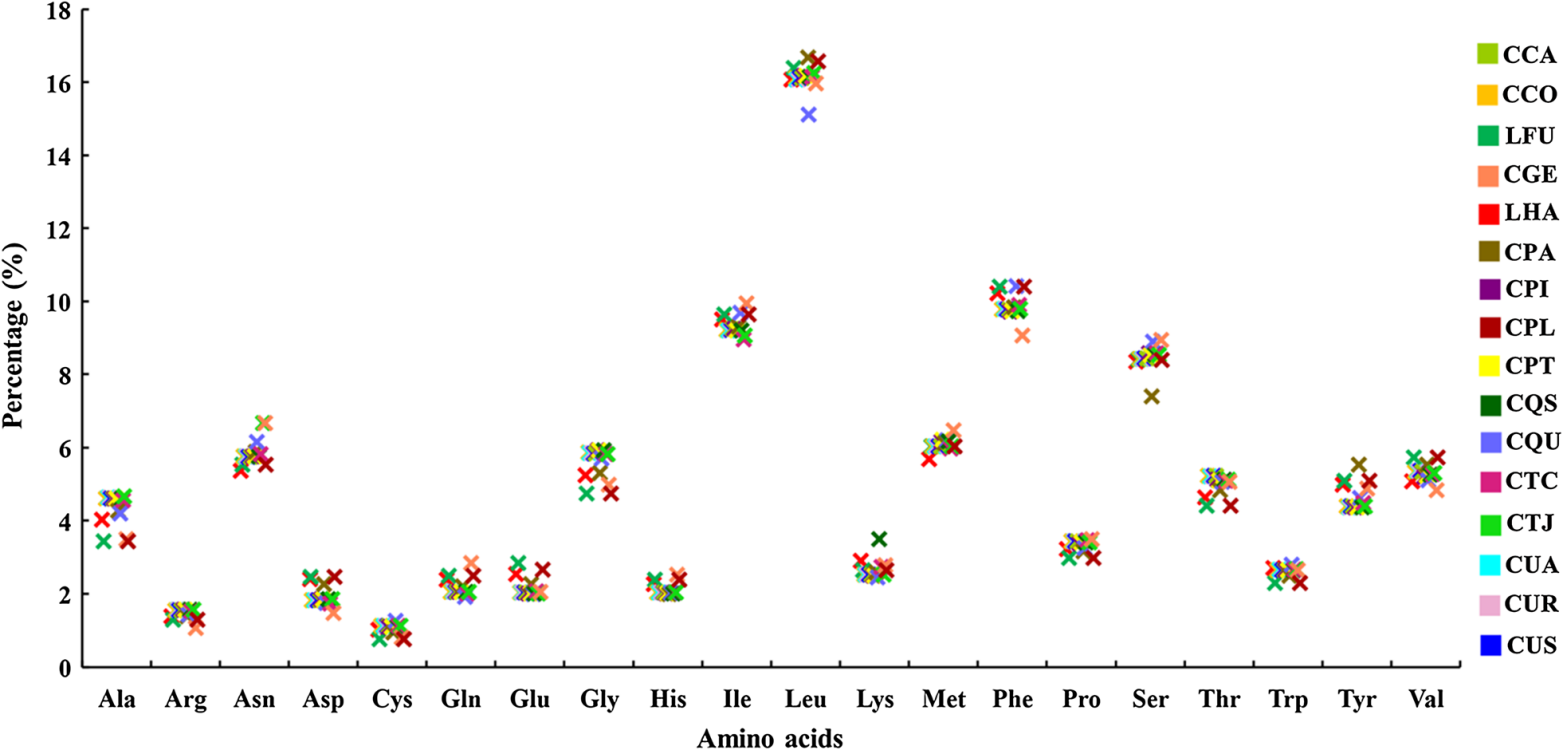
Frequency percentage of each of the 20 amino acids coded in the 16 *Culex* and *Lutzia* mt genomes. *Abbreviations*: CCA, *Cx. camposi*; CCO, *Cx. coronator*; LFU, *Lt. fuscanus*; CGE, *Cx. gelidus*; LHA, *Lt. halifaxia*; CPA, *Cx. p. pallens*; CPI, *Cx. p. pipiens*; CPL, *Cx. pallidothorax*; CPT, *Cx. pipiens* TU; CQS, *Cx. quinquefasciatus* USA; CQU, *Cx. quinquefasciatus*; CTC, *Cx. tritaeniorhynchus* CQ; CTJ, *Cx. tritaeniorhynchus* JS; CUA, *Cx. usquatissimus* AC; CUR, *Cx. usquatissimus* RO; CUS, *Cx. usquatus*

The RSCU values of the 16 investigated mt genomes are presented in Additional file 2: Table S2. In *Lt. fuscanus* and *Lt. halifaxia*, UUA is the most frequently used codon, followed by CGA, GGA and UCU, whereas CCG and ACG are rarely used, and CGC is not used. In *Cx. pallidothorax*, UUA is the most frequently used codon, followed by CGA, UCU and GGA, whereas CCG, ACG and CGC are not used. Among the 16 investigated mt genomes, UUA is the most frequently used codon, followed by CGA, GGA and UCU, whereas CGC, CCG and ACG are rarely used. Among the 16 investigated mt genomes, a total of 20 different amino acids are encoded, and the amino acid Leu has the highest frequency (16.33%), whereas Cys has the lowest (1.05%) (Fig. 3).

### Transfer RNAs, ribosomal RNAs and the CR

Twenty-two tRNAs were identified in the *Lt. halifaxia*, *Lt. fuscanus* and *Cx. pallidothorax* mt genomes; their secondary structures are presented in Additional file 3: Figure S1. The length of the tRNAs varies from 64 (*trnA*) to 74 bp (*trnN*) among the three mt genomes (Tables 2, 3, 4). Most of the tRNAs can be folded as a typical cloverleaf structure, except for *trnS*2, whose DHU arm simply forms an 11-nucleotide loop (Additional file 3: Figure S1). A total of 27 mismatched base pairs were detected in *Lt. halifaxia* tRNAs, 18 of which are UG pairs, and the remaining nine pairs include three AC pairs, three UU pairs, two AA pairs and one GA pair. Twenty-one mismatched base pairs were observed in *Lt. fuscanus* tRNAs, including 17 UG pairs, two AA pairs and two UU pairs. There are 23 mismatched base pairs in *Cx. pallidothorax* tRNAs, including 18 UG pairs, three AG pairs, one UU pair and one UG pair.

In the three newly sequenced mt genomes, two rRNAs (*rrnL* and *rrnS*) are located between *trnL*2 and *trnV*, and between *trnV* and CR, respectively. The length of the rRNAs is 2134 bp, with an AT content of 82.61% in *Lt. halifaxia*; 2126 bp, with an AT content of 82.78% in *Lt. fuscanus*; and 2128 bp, with an AT content of 82.08% in *Cx. pallidothorax*.

**Fig. 4:**
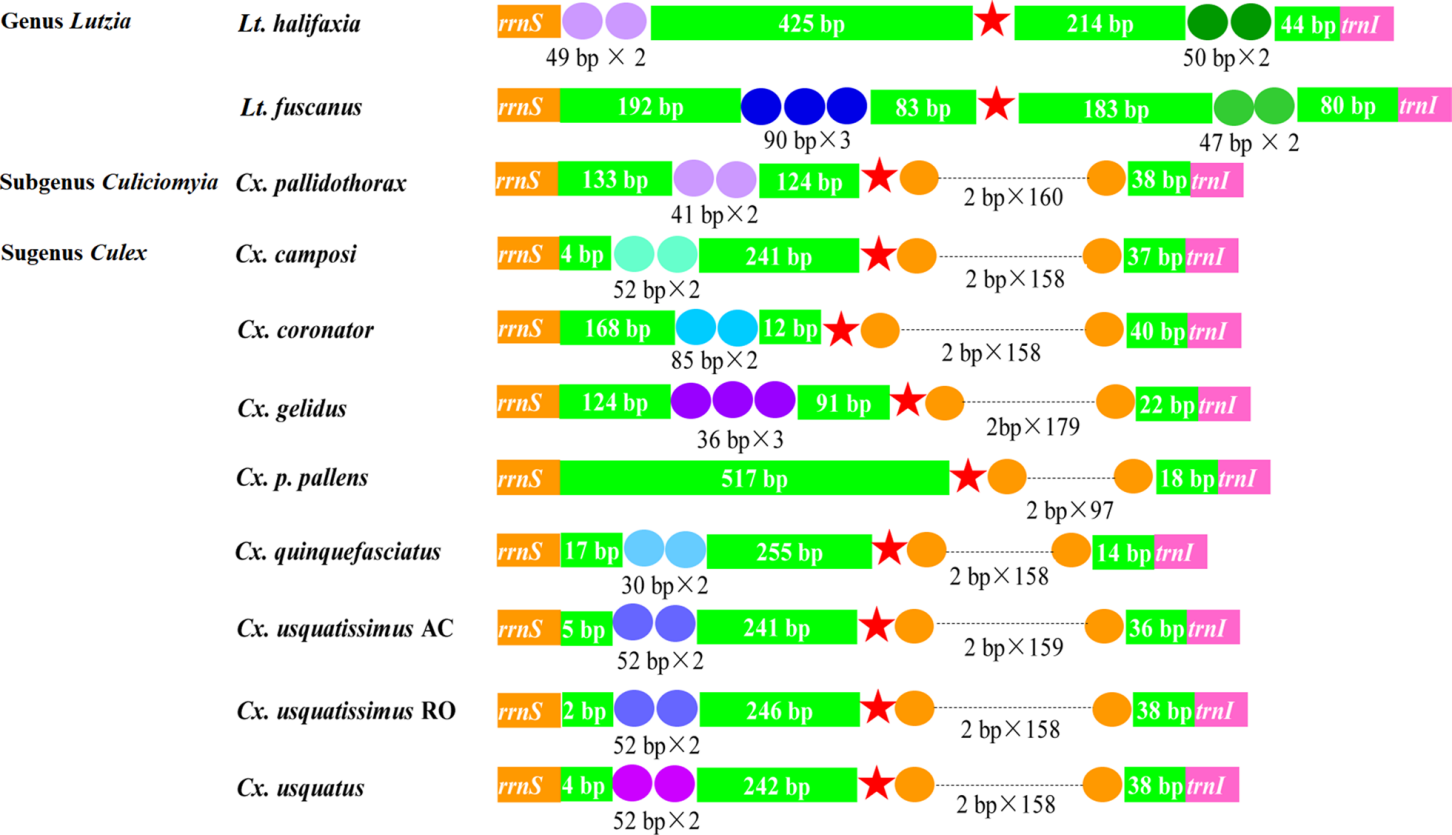
Conservative stretches of CRs in 11 *Culex* and *Lutzia* mt genomes with complete CR sequences. The ellipses filled with different colors indicate regions with different tandem repeat units (repeat number and unit bp length showing beneath the corresponding ellipses), the pentagrams denote regions with 17–20 repeats of nucleotide T, and the green-filled boxes demonstrate the non-repeat regions with the sequence length marked inside

The CR is located between *rrnS* and *trnI* and shows the highest AT content (88.88% in *Lt. halifaxia*, followed by 89.78% in *Lt. fuscanus* and 87.11% in *Cx. pallidothorax*) (Additional file 1: Table S1). The length of the CRs of *Lt. halifaxia*, *Lt. fuscanus* and *Cx. pallidothorax* are 899, 921 and 724 bp, respectively. For the nine *Culex* and two *Lutzia* mt genomes with known CRs, the CR lengths vary from 704 bp in *Cx. quinquefasciatus* to 920 bp in *Lt. fuscanus*, and their AT content ranges from 87.11% in *Cx. pallidothorax* to 90.58% in *Cx. gelidus*. The length of the CR in *Lt. halifaxia* and *Lt. fuscanus* is 174 to 216 bp greater than the other nine CRs (Table 1). We also detected a 49-bp repeat unit, a poly-T stretch of 17 bp and a 50-bp repeat unit in *Lt. halifaxia*; a 90-bp repeat unit, a poly-T stretch of 18 bp and a 47-bp repeat unit in *Lt. fuscanus*; and a 41-bp repeat unit, a poly-T stretch of 18 bp and a 320-bp microsatellite-like dinucleotide repeat region [(TA)n stretch] in *Cx. pallidothorax* (Fig. 4).

Assessment of the CR of nine *Culex* and two *Lutzia* mt genomes identified four types of repeat units, and the structure of four types of repeat units are conservative along the taxonomic taxa (Fig. 4). The first repeat unit is 17–20 bp of poly-T tract, which is located in the central part of the CR and exists in all these mt genomes. The second is a 30–90 bp sequence with 2–3 repeats; these sequences are all situated nearby *rrnS*, vary among species and occur in all species but *Cx. p. pallens*, in which the repeat unit might have been lost during evolution. The third is a 47–50 bp sequence with two repeats; these two sequences are situated proximal to *trnI* and vary and exist only in two species in the genus *Lutzia*. The fourth is a microsatellite-like TA sequence ([TA(A)]n stretch) consisting of 97–179 repeats, which is also situated close to *trnI* and exists in all nine species in the subgenera *Culiciomyia* and *Culex*.

### Phylogenetic relationships

**Fig. 5:**
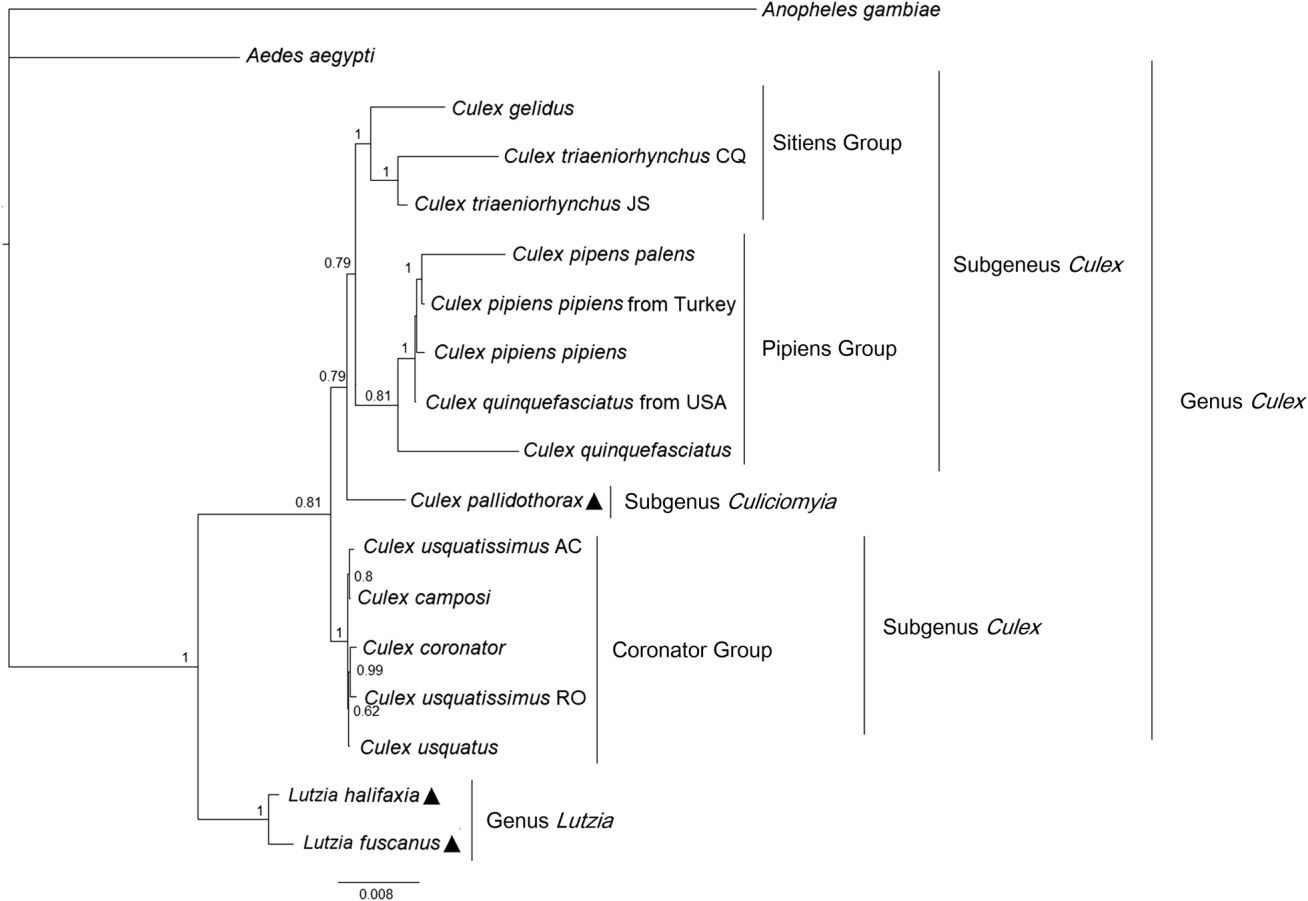
Phylogenetic relationships of 16 mt genomes based on nucleotide sequences of 13 protein-coding genes. The tree was constructed using BI method, and numbers at the nodes are Bayesian posterior probabilities. The newly sequenced mt genomes of three species are indicated by triangles. The GenBank accession numbers of mt genome sequences of the species are listed in Table 1

The best-fit model chosen for each gene and the resulting phylogenetic tree from the BI analysis are provided in Additional file 4: Table S3 and Fig. 5, respectively. The Bayesian topology shows *Lutzia* as the sister taxon of *Culex* spp. with a maximum posterior probability (pp = 1.0). Inside the clade composed by *Culex* spp., the monophyly of the Sitiens and Coronator groups was strongly supported (pp = 1.0), whereas the monophyletic status of the Pipiens Group was not supported (pp = 0.81). *Culex pallidothorax* was resolved sister to the clade compounded of Sitiens Group + Pipiens Group but the support was poor (pp = 0.79). Within the Sitiens group, *Culex gelidus* was a placed as sister species to *Culex tritaeniorhynchus* with high posterior probability. On the other hand, internal relationships of the Coronator Group were poorly resolved: one individual of *Culex usquatissimus* (AC) was placed as sister to *Culex camposi* (pp = 0.8) and the other (*Culex usquatissimus* RO) was sister to *Culex coronator*. The placement of *Culex usquatus* was weakly supported (0.62 pp). Similarly, in the Pipiens Group, clustering of one individual *Culex quinquefasciatus* within the clade of *Culex pipiens* specimens was strongly supported (1.0 pp).

## Discussion

### General characteristics of 16 *Culex* spp. mt genome sequences

Among the 16 mt genome sequences (including the three newly sequenced) of species in the genera *Culex* and *Lutzia*, 11 complete sequences were 15,573 to 15,803 bp in length, with variations mainly occurring in the CR, similar to that earlier reported in insects [15, 37, 38]. These 16 *Culex* mt genomes include 37 genes (13 PCGs, 22 tRNAs and two rRNAs) with a similar gene arrangement as those reported in other mosquito genera [19]. The nucleotide composition is biased toward AT (77.40–78.50%), with A being the most favored nucleotide (39.11–39.70%) and G as the least favored (9.10–9.44%), and with an average AT-skew value of 0.0107 and an average GC-skew value of -0.1572, features also similar to those previously reported in insects [13, 14, 19, 20, 37, 38].

Among the 16 *Culex* mt genomes, 13 PCGs showed variations in total nucleotide length ranging from 11,188 to 11,234 bp, with ATN being the most frequently used start codon, followed by TCG, and TAA being the most frequently used stop codon, followed by TA and T. The PCGs of most other mosquito species are also predicted to mainly use ATN as the start codon and TAA as the stop codon [19]. The incomplete stop codons are common in insect mt genomes [13, 14, 17, 19, 20, 37, 38] and the complete termination codon is thought to be created by post-transcriptional polyadenylation [39]. UUA is the most frequently used codon, followed by CGA, GGA and UCU, whereas CGC, CCG and ACG are rarely used, which is consistent with the observed higher AT content in these mt genomes. This phenomenon has also been observed in the mt genome of another mosquito species, *Anopheles minimus* [40]. The amino acids encoded by the codons ending with U or A are overused, with Leu being the most frequently encoded amino acid (16.33%) and Cys as the least frequently used amino acid (1.05%), which is also similar to that reported in some *Anopheles* mt genomes [19, 40, 41]. In terms of the *trnS*2 of three newly sequenced mt genomes, the DHU arm is not formed, which is similar to other *Culex* species [20–23] and metazoans [42]. The location of the two rRNAs is the same as in other Dipteran mt genomes [43].

We identified four types of repeat units in the nine *Culex* and two *Lutzia* mt genomes with complete CR sequences. Among the four types, the poly-T stretch and [TA(A)]n stretches have also been found in other insect species [44–47]. The poly-T stretch is highly conserved in insects, and it is thought to contain several regulatory elements, including the origin of replication and transcription [45]. The [TA(A)]n stretch exists in all nine mt genomes in the subgenera *Culiciomyia* and *Culex* investigated in the present study and in some other insect species [43], and it does not in two species in the genus *Lutzia* as determined in this study and in some other insect species, which suggest multiple evolutionary origins. Two other types of repeat units have not been found in other species; however, three other types of repeat units were identified in other insects, namely a highly conserved stem-and-loop structure, a G(A)nT structure and a G+A-rich stretch, which were not detected in the genus *Culex*. The repeat units are relatively conserved and thus may be utilized in phylogenetic reconstruction.

### Evolutionary relationships and taxonomy

The Coronator and Sitiens groups each form a unique clade with a posterior probability of 1, whereas the Pipiens group is poorly supported with a posterior probability of 0.82. These results support the earlier results of phylogenetic studies using mt genome sequences [21–23]. Morphologically, the *Cx. pallidothorax* has been classified within subgenus *Culiciomyia* [1, 24], whereas phylogenetic analysis based on *cox*1 sequences has shown that this species belongs to the subgenus *Culex*, with a low bootstrap support of 11% [11]. The phylogenetic analysis conducted in the present study indicates that *Cx. pallidothorax* belongs to the subgenus *Culex* and is sister of the groups Sitiens and Pipiens albeit with poor support. Additionally, the species has three types of repeat units, which is similar to that observed in species of the subgenus *Culex*. It appears that the taxonomic status of *Cx. pallidothorax* is doubtful and needs to be elucidated. In order to enlighten the position of *Culiciomyia* as subgenus, further analyses will be necessary using additional species.

Whether *Lutzia* should be considered as a genus or subgenus has long remained controversial. Morphological taxonomy identifies it as a genus [2, 4] or subgenus [3], whereas a phylogenetic analysis based on the morphological characteristics of larvae and adults has placed it outside the clade comprising the genus *Culex*. Molecular phylogenetic analysis using ITS1 and ITS2, including 14 species in the four subgenera of the genus *Culex*, showed that *Lutzia tigripes* was placed at the base of subgenus *Culex* (including three species in the Pipiens group and one species in the Sitiens group) [7]. Another analysis that also used ITS2, including 17 Neotropical species from five subgenera of genus *Culex*, classified *Lutzia* under subgenus *Culex* (including one species in the Pipiens group and two species in the Coronator group) [9]. The *cox*1-based analysis of 17 species from five subgenera of genus *Culex* (including one species in *Lutzia*, one species in the Pipiens group, and two species in the Coronator group in subgenus *Culex*) showed *Lutzia* as the sister taxon of the clade composed by the subgenera *Culex* + *Phenacomyia* [8]. Our phylogenetic analysis that included two *Lutzia* spp. (*Lt. halifaxia* and *Lt. fuscanus*) indicated *Lutzia* as a monophyletic entity and supports its original generic status. In the present study, the two *Lutzia* species have a 47–50-bp sequence with two repeats in the CR, which was not detected in other species. In addition, the two repeats lack the [TA(A)]n stretch, which is present in all other *Culex* species investigated. The assessment of features of the repeat units in the CR also supports the monophyly of this taxon.

## Conclusions

The present study sequenced and analyzed the complete mt genomes of *Lt. halifaxia*, *Lt. fuscanus* and *Cx. pallidothorax* and assessed the general characteristics and phylogenetic relationships of all known 16 mt genome sequences in the genera *Culex* and *Lutzia*. *Culex* spp. mt genomes share the same gene arrangement as other mosquito species, and variations in length mainly involve the CR. The repeat units of the CR are relatively conserved and provide information that may be utilized in establishing the phylogeny of *Culex* and *Lutzia*. The Coronator and Sitiens groups are each monophyletic, whereas the monophyletic status of the Pipiens Group was not supported. The taxonomic status of subgenus *Culiciomyia* has yet to be elucidated using additional species. Both phylogenetic analysis and repeat unit features of the CR show that *Lutzia* is a characteristic monophyletic group at the generic level. To our knowledge, this is first comprehensive review of the mt genome sequences and taxonomic assessment based on mt genome sequences of species in the genera *Culex* and *Lutzia*.

## Additional files

**Additional file 1: Table S1.** Composition and skewness of 16 *Culex* mt genomes.

**Additional file 2: Table S2.** Relative synonymous codon usage (RSCU) in the 16 *Culex* mt genomes.

**Additional file 3: Figure S1.** Predicted secondary structures of 22 tRNAs in the mt genomes of *Lt. fuscanus* (**a**), *Lt. halifaxia* (**b**) and *Cx. pallidothorax* (**c**).

**Additional file 4: Table S3.** Best-fit models chosen under Akaike information criterion by Modeltest for each of the 13 PCGs.

## Data Availability

All data are available as tables and figures in the main document and its additional files. The GenBank accession numbers for the three mt genomes produced in the present study are MH316119 (*Lt. halifaxia*), MH316118 (*Lt. fuscanus*) and KY400104 (*Cx. pallidothorax*).

## Abbreviations

mt genome: mitochondrial genome
PCGs: protein-coding genes
rRNAs: ribosomal RNA genes
tRNAs: transfer RNA genes
CR: control region
RSCU: relative synonymous codon usage
ML: maximum likelihood
BI: Bayesian inference
TR: tandem repeats

## References

[1] Harbach RE (2011). Classification within the cosmopolitan genus *Culex* (Diptera: Culicidae): The foundation for molecular systematics and phylogenetic research. Acta Trop..

[2] Theobald FV (1903). A monograph of Culicidae or mosqutoes.

[3] Edwards FW. Genera insectorum. Diptera, Fam. Culicidae. Fasc. 194. Brussels: Desmet-Verteneuil; 1932.

[4] Tanaka K (2003). Studies on the pupal mosquitoes of Japan Genus *Lutzia*, with establishment of two new subgenera, *Metalutzia* and *Insulalutzia* (Diptera: Culicidae). Jpn J Syst Entomol..

[5] Navarro JC, Liria J (2000). Phylogenetic relationships among eighteen neotropical Culicini species. J Am Mosq Control Assoc..

[6] Harbach RE, Kitching IJ, Culverwell CL, Culverwell CL, Dubois J, Linton YM (2012). Phylogeny of mosquitoes of tribe Culicini (Diptera: Culicidae) based on morphological diversity. Zool Scr..

[7] Miller BR, Crabtree MB, Savage HM (1996). Phylogeny of fourteen *Culex* mosquito species, including the *Culex pipiens* complex, inferred from the internal transcribed spacers of ribosomal DNA. Insect Mol Biol..

[8] Demari-Silva B, Vesgueiro FT, Sallum MAM, Marrelli MT (2011). Taxonomic and phylogenetic relationships between species of the genus *Culex* (Diptera: Culicidae) from Brazil inferred from the cytochrome c oxidase I mitochondrial gene. J Med Entomol..

[9] Vesgueiro FT, Demari-Silva B, Malafronte RS, Sallum MA, Marrelli MT (2011). Intragenomic variation in the second internal transcribed spacer of the ribosomal DNA of species of the genera *Culex* and *Lutzia* (Diptera: Culicidae). Mem Inst Oswaldo Cruz..

[10] Edwards FW (1921). A revision of the mosquitos of the Palaearctic Region Bull Entomol Res..

[11] Wang G, Li CX, Guo XX, Xing D, Dong YD, Wang ZM (2012). Identifying the main mosquito species in China based on DNA barcoding. PLoS ONE..

[12] Balaban RS, Nemoto S, Finkel T (2005). Mitochondria, oxidants, and aging. Cell..

[13] Salvato P, Simonato M, Battisti A, Negrisolo E (2008). The complete mitochondrial genome of the bag-shelter moth *Ochrogaster lunifer* (Lepidoptera, Notodontidae). BMC Genomics..

[14] Dai LS, Qian C, Zhang CF, Wang L, Wei GQ, Li J (2015). Characterization of the complete mitochondrial genome of *Cerura menciana* and comparison with other Lepidopteran insects. PLoS ONE..

[15] Wang YY, Liu XY, Garzón-Ordunac IJ, Winterton SL, Yan Y, Aspöck U (2016). Mitochondrial phylogenomics illuminates the evolutionary history of Neuropterida. Cladistics..

[16] Li Q, Wei SJ, Tang P, Wu Q, Shi M, Sharkey MJ (2016). Multiple lines of evidence from mitochondrial genomes resolve phylogenetic relationships of parasitic wasps in Braconidae. Genome Biol Evol..

[17] Breeschoten T, Doorenweerd C, Tarasov S, Vogler AP (2016). Phylogenetics and biogeography of the dung beetle genus *Onthophagus*, inferred from mitochondrial genomes. Mol Phylogenet Evol..

[18] Song N, An SH, Yin XM, Cai WZ, Li H (2016). Application of RNA-seq for mitogenome reconstruction, and reconsideration of long-branch artifacts in Hemiptera phylogeny. Sci Rep..

[19] Hao YJ, Zou YL, Ding YR, Xu WY, Yan ZT, Li XD (2017). Complete mitochondrial genomes of *Anopheles stephensi* and *An dirus* and comparative evolutionary mitochondriomics of 50 mosquitoes. Sci Rep..

[20] Behura SK, Lobo NF, Haas B, Debruyn B, Lovin DD, Shumway MF (2011). Complete sequences of mitochondria genomes of *Aedes aegypti* and *Culex quinquefasciatus* and comparative analysis of mitochondrial DNA fragments inserted in the nuclear genomes. Insect Biochem Mol..

[21] Demari-Silva B, Foster PG, Oliveira TMPD, Bergo ES, Sanabani SS, Pessoa R (2015). Mitochondrial genomes and comparative analyses of *Culex camposi*, *Culex coronator*, *Culex usquatus* and *Culex usquatissimus* (Diptera: Culicidae), members of the Coronator group. BMC Genomics..

[22] Luo QC, Hao YJ, Meng FX, Li TJ, Ding YR, Hua YQ, Chen B (2016). The mitochondrial genomes of *Culex pipiens pallens* and *Culex tritaeniorhynchus* (Diptera: Culicidae) and comparison analysis with two other *Culex* species. Parasit Vectors..

[23] Sun L, Fu WB, Yan ZT, Li TJ, Ding YR, Chen B (2017). Sequencing and analysis of the complete mitochondrial genome of *Culex gelidus* (Diptera: Culicidae). Mit DNA Part B..

[24] Lu BL. Fauna Sinica. Insecta. Diptera: Culicidae 1. Vol. 8. Beijing, China: Science Press; 1997.

[25] Sun L, Fu WB, Yan ZT, Chen B (2018). Molecular phylogenetic relationships among 40 species (subspecies) in the genus *Culex* from China (Diptera: Culicidae). Acta Entomol Sinica..

[26] Zhang NX, Zhang YJ, Yu G, Chen B (2013). Structure characteristics of the mitochondrial genomes of Diptera and design and application of universal primers for their sequencing. Acta Entomol Sinica..

[27] Thompson JD, Gibson TJ, Plewniak F, Jeanmougin F, Higgins DG (1997). The CLUSTAL X Windows interface: flexible strategies for multiple sequence alignment aided by quality analysis tools. Nucleic Acids Res..

[28] Lowe TM, Eddy SR (1997). tRNAscan-SE: a program for improved detection of transfer RNA genes in genomic sequence. Nucleic Acids Res..

[29] Tamura K, Peterson D, Peterson N, Stecher G, Nei M (2011). MEGA5: molecular evolutionary genetics analysisusing maximum likelihood, evolutionary distance, and maxi-mum parsimony methods. Mol Biol Evol..

[30] Perna NT, Kocher TD (1995). Patterns of nucleotide composition at fourfold degenerate sites of animal mitochondrial genomes. J Mol Evol..

[31] Grant JR, Stothard P (2008). The CGView Server: a comparative genomics tool for circular genomes. Nucleic Acids Res..

[32] Mikrajuddin A, Khairurrijal A (2009). A simple method for determining surface porosity based on SEM images using Origin Pro software. Indonesian J Phys..

[33] Zuker M (2003). Mfold web server for nucleic acid folding and hybridization prediction. Nucleic Acids Res..

[34] Ronquist F, Teslenko M, van derMark P, Ayres DL, Darling A, Höhna S (2012). MrBayes 3.2: efficient Bayesian phylogenetic inference and model choice across a large model space. Syst Biol..

[35] Abascal F, Zardoya R, Telford MJ (2010). TranslatorX: multiple alignment of nucleotide sequences guided by amino acid translations. Nucl Acids Res..

[36] Posada D, Crandall KA (1998). MODELTEST: testing the model of DNA substitution. Bioinformatics..

[37] Chen YH, Huang DY, Wang YL, Zhu CD, Hao JS (2014). The complete mitochondrial genome of the endangered Apollo butterfly, *Parnassius apollo*, (Lepidoptera: Papilionidae) and its comparison to other Papilionidae species. J Asia-Pac Entomol..

[38] Wang Y, Liu X, Yang D (2014). The first mitochondrial genome for caddisfly (Insecta: Trichoptera) with phylogenetic implications. Int J Biol Sci..

[39] Ojala D, Montoya J, Attardi G (1981). tRNA punctuation model of RNA processing in human mitochondria. Nature..

[40] Hua YQ, Ding YR, Yan ZT, Si FL, Luo QC, Chen B (2016). The complete mitochondrial genome of *Anopheles minimus* (Diptera: Culicidae) and the phylogenetics of known *Anopheles* mitogenomes. Insect Sci..

[41] Hua YQ, Yan ZT, Fu WB, He QY, Zhou Y, Chen B (2015). Sequencing and analysis of the complete mitogenome in *Anopheles culicifacies* species B (Diptera: Culicidae). Mit DNA Part A..

[42] Negrisolo E, Babbucci M, Patarnello T (2011). The mitochondrial genome of the ascalaphid owlfly *Libelloides macaroniusand* comparative evolutionary mitochondriomics of neuropterid insects. BMC Genomics..

[43] Zhang NX, Yu G, Li TJ, He QY, Zhou Y, Si FL (2015). The complete mitochondrial genome of *Delia antiqua* and its implications in dipteran phylogenetics. PLoS ONE..

[44] Zhang DX, Hewitt GM (1997). Insect mitochondrial control region: a review of its structure, evolution and usefulness in evolutionary studies. Biochem Syst Ecol..

[45] Sun WY, Xu DL, Chen HX, Shi W, Sundberg P, Strand M, Sun SC (2014). Complete mitochondrial genome sequences of two parasitic/commensal nemerteans, *Gononemertes* parasite and *Nemertopsis tetraclitophila* (Nemertea: Hoplonemertea). Parasit Vectors..

[46] Spanos L, Koutroumbas G, Kotsyfakis M, Louis C (2000). The mitogenome of the Mediterranean fruit fly. Ceratitis capitata. Insect Mol Biol..

[47] Zhou Z, Huang Y, Shi F, Ye H (2009). The complete mitogenome of *Deracantha onos* (Orthoptera: Bradyporidae). Mol Biol Rep..

