# Supplementary material for: The complete mt genomes of Lutzia halifaxia, Lt. fuscanus and Culex pallidothorax (Diptera: Culicidae) and comparative analysis of 16 Culex and Lutzia mt genome sequences
Source: Parasit Vectors. 2019 Jul 26;12:368. doi: 10.1186/s13071-019-3625-2 (PMC6660957; doi:10.1186/s13071-019-3625-2)
Supplement: Supplementary file 1 — Additional file 1: Table S1. Composition and skewness of 16 Culex mt genomes. [file 13071_2019_3625_MOESM1_ESM.pdf]

Table S1. Composition and skewness of 16 *Culex* and *Lutzia* mt genomes.

Genus/Subgenus	Species	A%	T%	G%	C%	A+T%	AT-Skew	GC-Skew
Mitogenomes (without CR)								
Lutzia	*Lt. halifaxia*	39.28	38.68	9.30	12.74	77.96	0.0078	-0.1613
	Lt . fuscanus	39.70	38.70	9.10	12.60	78.40	0.0128	-0.1559
Culiciomyia	*Cx. pallidothorax*	39.70	38.80	9.10	12.70	78.50	0.0078	-0.1651
Culex	*Cx. coronator*	39.31	38.72	9.32	12.66	78.02	0.0075	-0.1520
	Cx. usquatissimus AC	39.29	38.72	9.36	12.63	78.01	0.0073	-0.1488
	Cx. usquatissimus RO	39.28	38.72	9.35	12.65	78.00	0.0072	-0.1499
	Cx. usquatu	39.35	38.75	9.26	12.64	78.10	0.0076	-0.1540
	Cx. camposi	39.38	38.78	9.28	12.56	78.16	0.0076	-0.1504
	Cx. p. pipiens	39.36	38.27	9.44	12.93	77.63	0.0140	-0.1562
	Cx. pipiens TU	39.40	38.31	9.40	12.89	77.71	0.1430	-0.1568
	Cx. p. pallens	39.39	38.28	9.41	12.93	77.66	0.1291	-0.1577
	Cx. quinquefasciatus	39.20	38.20	9.40	13.20	77.40	0.1393	-0.1681
	Cx. quinquefasciatus USA	39.37	38.29	9.43	12.90	77.67	0.0844	-0.1555
	Cx. tritaeniorhynchus CQ	39.11	38.46	9.40	13.03	77.57	0.1162	-0.1619
	Cx. tritaeniorhynchus JS	39.20	38.30	9.40	12.10	77.50	0.1282	-0.1644
	Cx. gelidus	39.70	38.70	9.10	12.50	78.40	0.1154	-0.1574
Average						77.92	0.0107	-0.1572
PCGs								
Lutzia	*Lt. halifaxia*	32.74	44.14	12.15	10.97	76.88	-0.1483	0.0508
	Lt. fuscanus	32.87	44.31	11.86	10.96	77.18	-0.1483	0.0391
Culiciomyia	*Cx. pallidothorax*	32.67	44.45	12.02	10.86	77.12	-0.1527	0.0506
Culex	*Cx. coronator*	32.45	44.62	12.16	10.77	77.08	-0.1578	0.0606
	Cx. usquatissimus AC	32.51	44.56	12.09	10.85	77.07	-0.1564	0.0540
	Cx. usquatissimus RO	32.48	44.56	12.14	10.82	77.04	-0.1567	0.0571
	Cx. usquatu	32.55	44.60	12.08	10.77	77.16	-0.1562	0.0573
	Cx. camposi	32.56	44.63	12.06	10.75	77.19	-0.1563	0.0574
	Cx. p. pipiens	32.11	44.35	12.47	11.07	76.47	-0.1600	0.0596
	Cx. pipiens TU	32.24	44.37	12.40	10.98	76.61	-0.1584	0.0606
	Cx. p. pallens	32.27	44.29	12.44	11.01	76.55	-0.1570	0.0607
	Cx. quinquefasciatus	32.07	44.25	12.51	11.17	76.32	-0.1596	0.0568
	Cx. quinquefasciatus USA	32.21	44.35	12.43	11.01	76.56	-0.1585	0.0605
	Cx. tritaeniorhynchus CQ	32.36	44.15	12.31	11.19	76.50	-0.1541	0.0478
	Cx. tritaeniorhynchus JS	32.20	44.16	12.35	11.28	76.37	-0.1566	0.0452
	Cx. gelidus	32.81	44.74	11.89	10.56	77.55	-0.1537	0.0591
Average						76.85	-0.1557	0.0548
tRNAS								
Lutzia	*Lt. halifaxia*	39.76	39.96	11.39	8.89	79.72	-0.0025	0.1229
	Lt. fuscanus	40.04	40.04	11.34	8.58	80.08	0	0.1390
Culiciomyia	*Cx. pallidothorax*	39.68	39.54	11.67	9.11	79.22	0.0017	0.1234
Culex	*Cx. coronator*	39.33	39.73	11.82	9.12	79.06	-0.0051	0.1287
	Cx. usquatissimus AC	39.13	39.73	11.96	9.18	78.86	-0.0077	0.1312

	Cx. usquatissimus RO	39.36	39.74	11.79	9.11	79.10	-0.0049	0.1282
	Cx. usquatu	39.16	39.44	12.00	9.40	78.60	-0.0036	0.1212
	Cx. camposi	39.34	39.74	11.81	9.11	79.08	-0.0051	0.1290
	Cx. p. pipiens	40.61	38.37	9.29	11.73	78.98	0.0283	-0.1161
	Cx. pipiens TU	40.54	38.37	9.36	11.73	78.92	0.0275	-0.1125
	Cx. p. pallens	40.01	38.93	11.74	9.31	78.95	0.0137	0.1154
	Cx. quinquefasciatus	40.56	38.10	9.61	11.72	78.66	0.0312	-0.0990
	Cx. quinquefasciatus USA	40.79	38.48	9.35	11.38	79.27	0.0291	-0.0980
	Cx. tritaeniorhynchus CQ	39.06	39.73	12.15	9.06	78.79	-0.0085	0.1456
	Cx. tritaeniorhynchus JS	39.24	39.78	11.74	9.23	79.02	-0.0069	0.1197
	Cx. gelidus	39.89	39.67	11.53	8.91	79.56	0.0027	0.1280
Average						79.12	0.0056	0.0692
rRNAs								
Lutzia	*Lt. halifaxia*	40.25	42.36	11.29	6.09	82.61	-0.0255	0.2992
	Lt. fuscanus	40.26	42.52	11.10	6.11	82.78	-0.0273	0.2896
Culiciomyia	*Cx. pallidothorax*	40.20	41.88	11.45	6.47	82.08	-0.0204	0.2779
Culex	*Cx. coronator*	40.21	41.95	11.39	6.45	82.16	-0.0212	0.2770
	Cx. usquatissimus AC	40.16	41.95	11.44	6.45	82.11	-0.0218	0.2789
	Cx. usquatissimus RO	40.11	42.00	11.49	6.40	82.11	-0.0229	0.2842
	Cx. usquatu	40.21	42.04	11.39	6.36	82.25	-0.0223	0.2838
	Cx. camposi	40.30	42.14	11.30	6.26	82.44	-0.0223	0.2869
	Cx. p. pipiens	42.21	40.04	6.33	11.43	82.25	0.0264	-0.2872
	Cx. pipiens TU	42.26	40.08	6.28	11.38	82.34	0.0264	-0.2888
	Cx. p. pallens	40.18	42.28	11.27	6.27	82.46	-0.0255	0.2853
	Cx. quinquefasciatus	42.16	40.15	6.32	11.37	82.31	0.0244	-0.2857
	Cx. quinquefasciatus USA	42.21	40.08	6.33	11.38	82.29	0.0258	-0.2853
	Cx. tritaeniorhynchus CQ	40.41	42.04	11.34	6.21	82.45	-0.0198	0.2926
	Cx. tritaeniorhynchus JS	41.35	41.12	8.08	9.45	82.47	0.0028	-0.0777
	Cx. gelidus	40.51	41.70	11.35	6.44	82.20	-0.0145	0.2761
Average						82.33	-0.0086	0.1192
CR								
Lutzia	*Lt. halifaxia*	43.49	45.38	4.56	6.56	88.88	-0.0213	-0.1800
	Lt. fuscanus	43.59	46.20	3.59	6.63	89.78	-0.0291	-0.2979
Culiciomyia	*Cx. pallidothorax*	38.66	48.46	3.78	9.10	87.11	-0.1125	-0.4130
Culex	*Cx. coronator*	41.63	47.72	3.18	7.47	89.35	-0.0681	-0.4026
	Cx. usquatissimus AC	41.19	47.99	3.61	7.21	89.18	-0.0762	-0.3333
	Cx. usquatissimus RO	41.14	48.34	3.32	7.20	89.47	-0.0805	-0.3684
	Cx. usquatu	41.72	47.71	3.34	7.23	89.43	-0.0669	-0.3684
	Cx. camposi	41.86	47.71	3.34	7.09	89.57	-0.0652	-0.3600
	Cx. p. pallens	41.10	47.66	3.75	7.50	88.76	-0.0739	-0.3333
	Cx. quinquefasciatus	40.06	48.44	3.84	7.67	88.49	-0.0947	-0.3333
	Cx. gelidus	40.58	50.00	3.32	6.09	90.58	-0.1040	-0.2941
Average						89.15	-0.0720	-0.3350
