# Supplementary material for: The complete mt genomes of Lutzia halifaxia, Lt. fuscanus and Culex pallidothorax (Diptera: Culicidae) and comparative analysis of 16 Culex and Lutzia mt genome sequences
Source: Parasit Vectors. 2019 Jul 26;12:368. doi: 10.1186/s13071-019-3625-2 (PMC6660957; doi:10.1186/s13071-019-3625-2)
Supplement: Supplementary file 2 — Additional file 2: Table S2. Relative synonymous codon usage (RSCU) in the 16 Culex mt genomes. [file 13071_2019_3625_MOESM2_ESM.pdf]

Table S2 Relative synonymous codon usage (RSCU) in the 16 *Culex* and *Lutzia* mt genomes.

Amino acid	Codons	RSCU															
		CCA	CCO	LFU	CGE	LHA	CPA	CPI	CPL	CPT	CQS	CQU	CTC	CTJ	CUA	CUR	CUS
Ala	GCU	2.74	2.84	2.43	2.27	2.47	2.74	2.74	2.47	2.74	2.74	2.92	2.80	2.8	2.82	2.82	2.77
	GCC	0.21	0.14	0.21	0.34	0.41	0.18	0.16	0.11	0.16	0.16	0.16	0.21	0.28	0.18	0.18	0.02
	GCA	0.98	0.98	1.32	1.36	0.93	0.92	0.98	1.25	0.98	0.98	0.82	0.92	0.87	0.95	0.95	0.89
	GCG	0.07	0.05	0.04	0.03	0.19	0.16	0.12	0.17	0.12	0.12	0.05	0.07	0.05	0.05	0.05	0.37
Arg	CGU	0.55	0.55	0.76	0.44	0.96	0.47	0.48	0.51	0.48	0.48	0.68	0.7	0.55	0.55	0.55	0.55
	CGC	0	0	0	0.11	0	0	0	0.09	0	0	0.08	0	0	0	0	0
	CGA	3.45	3.45	2.7	3.44	2.8	3.33	3.31	3.23	3.31	3.31	3.02	3.23	3.38	3.45	3.54	3.45
	CGG	0	0	0.54	0	0.24	0.2	0.21	0.17	0.21	0.21	0.23	0.07	0.07	0	0	0
Asn	AAU	1.89	1.87	1.84	1.61	1.73	1.81	1.8	1.87	1.8	1.8	1.82	1.83	1.85	1.89	1.89	1.87
	AAC	0.11	0.13	0.16	0.39	0.27	0.19	0.2	0.13	0.2	0.2	0.18	0.17	0.15	0.11	0.11	0.13
Asp	GAU	1.82	1.85	1.7	1.76	1.72	1.83	1.83	1.69	1.83	1.83	1.85	1.75	1.71	1.82	1.82	1.79
	GAC	0.18	0.15	0.3	0.24	0.28	0.17	0.17	0.31	0.17	0.17	0.15	0.25	0.29	0.18	0.18	0.21
Cys	UGU	1.95	1.9	1.89	1.63	1.75	1.90	1.95	1.79	1.95	1.95	1.7	2	1.95	1.86	1.86	1.95
	UGC	0.05	0.1	0.11	0.37	0.25	0.1	0.05	0.21	0.05	0.05	0.3	0	0.05	0.14	0.14	0.05
Gln	CAA	1.92	1.92	1.86	1.71	1.62	1.92	1.95	1.61	1.95	1.95	1.92	1.92	1.92	1.92	1.92	1.92
	CAG	0.08	0.08	0.14	0.29	0.38	0.08	0.05	0.39	0.05	0.05	0.08	0.08	0.08	0.08	0.08	0.08
Glu	GAA	1.97	1.97	1.7	1.71	1.46	1.92	1.97	1.47	1.97	1.97	1.89	1.92	1.95	1.97	1.97	1.97
	GAG	0.03	0.03	0.3	0.29	0.54	0.08	0.03	0.53	0.03	0.03	0.11	0.08	0.05	0.03	0.03	0.03
Gly	GGU	0.84	0.86	0.47	0.3	0.62	0.84	0.78	0.58	0.78	0.78	0.74	0.79	0.68	0.86	0.86	0.84
	GGC	0.02	0.05	0.02	0.03	0.06	0.06	0.05	0.18	0.05	0.04	0.11	0.02	0.07	0.02	0.02	0.02
	GGA	2.79	2.63	2.63	3.25	2.97	2.55	2.52	2.74	2.55	2.57	2.55	2.69	2.69	2.83	2.83	2.77
	GGG	0.35	0.46	0.61	0.3	0.54	0.55	0.65	0.49	0.62	0.62	0.6	0.50	0.55	0.29	0.29	0.37
His	CAU	1.71	1.68	1.73	0.03	1.67	1.70	1.68	1.68	1.68	1.68	1.63	1.58	1.53	1.71	1.63	1.63
	CAC	0.29	0.32	0.27	0.42	0.33	0.30	0.32	0.32	0.32	0.32	0.37	0.42	0.47	0.29	0.37	0.37
Ile	AUU	1.92	1.92	1.93	1.71	1.86	1.87	1.87	1.86	1.88	1.87	1.85	1.93	1.93	1.92	1.92	1.93
	AUC	0.08	0.08	0.07	0.29	0.14	0.13	0.13	0.14	0.12	0.13	0.15	0.07	0.07	0.08	0.08	0.07

Leu2 (UUR)	UUA	5.33	5.27	4.55	4.1	3.42	5.14	5.15	4.86	5.16	5.17	5.06	5.08	5.06	5.23	5.23	5.28
	UUG	0.09	0.12	0.3	0.3	0.56	0.21	0.2	0.14	0.2	0.19	0.21	0.24	0.22	0.11	0.11	0.1
Leu1 (CUN)	CUU	0.4	0.4	0.68	0.48	1.14	0.39	0.4	1.98	0.4	0.4	0.42	0.34	0.33	0.4	0.4	0.4
	CUC	0	0	0.06	0.25	0.15	0.01	0	0.15	0	0	0.03	0	0.01	0	0	0
	CUA	0.18	0.2	0.3	0.79	0.44	0.25	0.25	1.52	0.24	0.24	0.28	0.34	0.39	0.26	0.26	0.22
	CUG	0	0.01	0.11	0.1	0.29	0.01	0	0.34	0	0	0	0	0	0	0	0
Lys	AAA	1.38	1.43	1.75	1.63	1.68	1.53	1.78	1.59	1.51	1.51	1.45	1.47	1.38	1.34	1.34	1.36
	AAG	0.62	0.57	0.25	0.37	0.32	0.47	0.22	0.41	0.49	0.49	0.55	0.53	0.62	0.66	0.66	0.64
Met	AUA	1.8	1.8	1.74	1.83	1.62	1.81	1.51	1.74	1.8	1.79	1.77	1.79	1.79	1.77	1.77	1.8
	AUG	0.2	0.2	0.26	0.17	0.38	0.19	0.49	0.26	0.2	0.21	0.23	0.21	0.21	0.23	0.23	0.2
Phe	UUU	1.83	1.84	1.86	1.66	1.7	1.85	1.83	1.73	1.83	1.83	1.75	1.74	1.74	1.82	1.82	1.84
	UUC	0.17	0.16	0.14	0.34	0.3	0.15	0.17	0.27	0.17	0.17	0.25	0.26	0.26	0.18	0.18	0.16
Pro	CCU	2.34	2.31	2.09	2.03	2.02	2.32	2.29	2.82	2.27	2.27	2.21	2.26	2.33	2.31	2.31	2.31
	CCC	0.16	0.19	0.65	0.71	0.63	0.22	0.22	0.31	0.22	0.22	0.2	0.34	0.28	0.19	0.19	0.16
	CCA	1.5	1.47	1.2	1.19	1.3	1.46	1.49	0.86	1.51	1.48	1.55	1.4	1.39	1.47	1.47	1.53
	CCG	0	0.03	0.07	0.07	0.04	0	0	0	0	0.03	0.03	0	0	0.03	0.03	0
Ser1 (UCN)	UCU	2.54	2.52	2.92	1.89	2.71	2.64	2.7	1.83	2.69	2.71	2.49	2.63	2.72	2.52	2.52	2.54
	UCC	0.05	0.05	0.18	0.74	0.2	0.18	0.13	0.25	0.13	0.13	0.31	0.15	0.08	0.05	0.05	0.05
	UCA	2.54	2.55	2.14	2.13	2.35	2.08	2.17	1.89	2.16	2.18	1.81	2.25	2.26	2.55	2.55	2.54
	UCG	0.03	0.05	0.09	0.13	0.21	0.13	0.13	0.02	0.13	0.1	0.17	0.03	0.08	0.05	0.05	0.03
Ser2 (AGN)	AGU	1.83	1.83	1.23	1.2	1.25	1.78	1.77	2.1	1.81	1.78	1.57	1.63	1.74	1.83	1.83	1.83
	AGC	0.1	0.05	0.15	0.66	0.2	0.10	0.1	0.5	0.08	0.1	0.22	0.15	0.1	0.05	0.05	0.08
	AGA	0.91	0.94	1.2	1.01	0.82	1.04	1	1.08	1.01	1	1.04	1.1	1.03	0.94	0.94	0.94
	AGG	0	0	0.09	0.24	0.35	0.05	0	0.37	0	0	0.39	0.07	0	0	0	0
Thr	ACU	2.26	2.24	1.67	2	1.91	2.06	2.03	2.16	2.04	2.03	1.96	2.08	2.14	2.25	2.25	2.26
	ACC	0.02	0.04	0.31	0.25	0.38	0.08	0.08	0.16	0.08	0.08	0.17	0.13	0.1	0.02	0.02	0.02
	ACA	1.72	1.7	1.95	1.69	1.71	1.86	1.86	1.51	1.87	1.86	1.83	1.77	1.74	1.71	1.71	1.72
	ACG	0	0.02	0.07	0.06	0	0	0.02	0.18	0	0.02	0.04	0.02	0.02	0.02	0.02	0
Trp	UGA	1.83	1.83	1.23	1.91	1.88	1.92	1.9	1.92	1.92	1.9	1.92	1.98	2	1.83	1.83	1.83
	UGG	0.1	0.05	0.15	0.09	0.12	0.08	0.1	0.08	0.08	0.1	0.08	0.02	0	0.05	0.05	0.08
Tyr	UAU	0.91	0.94	1.2	1.66	1.66	1.82	1.83	1.82	1.83	1.83	1.79	1.83	1.87	0.94	0.94	0.94

	UAC	0	0	0.09	0.34	0.34	0.18	0.17	0.18	0.17	0.17	0.21	0.17	0.13	0	0	0
Val	GUU	1.89	1.91	1.8	1.74	1.89	2.04	2.06	2.04	2.07	2.06	1.96	2.02	1.94	1.88	1.88	1.9
	GUC	0	0	0.09	0.12	0.11	0.08	0.08	0.08	0.08	0.08	0.17	0.08	0.12	0.02	0.02	0
	GUA	2.03	2.01	1.98	2.05	1.54	1.76	1.75	1.76	1.74	1.76	1.77	1.82	1.84	2.02	2.02	2.02
	GUG	0.08	0.08	0.13	0.09	0.46	0.12	0.1	0.12	0.10	0.1	0.11	0.08	0.1	0.08	0.08	0.88

CCA: *Cx. camposi*, CCO: *Cx. coronator*, LFU: *Lt. fuscanus*, CGE: *Cx. gelidus*, LHA: *Lt. halifaxia*, CPA: *Cx. p. pallens*, CPI: *Cx. p. pipiens*, CPL: *Cx. pallidothorax*, CPT: *Cx. pipiens* TU, CQS: *Cx. quinquefasciatus* USA, CQU: *Cx. quinquefasciatus*, CTC: *Cx. tritaeniorhynchus* CQ, CTJ: *Cx. tritaeniorhynchus* JS, CUA: *Cx. usquatissimus* AC, CUR: *Cx. usquatissimus* RO, CUS: *Cx. usquatus.*
