# Supplementary material for: The complete mt genomes of Lutzia halifaxia, Lt. fuscanus and Culex pallidothorax (Diptera: Culicidae) and comparative analysis of 16 Culex and Lutzia mt genome sequences
Source: Parasit Vectors. 2019 Jul 26;12:368. doi: 10.1186/s13071-019-3625-2 (PMC6660957; doi:10.1186/s13071-019-3625-2)
Supplement: Supplementary file 3 — Additional file 3: Figure S1. Predicted secondary structures of 22 tRNAs in the mt genomes of Lt. fuscanus (a), Lt. halifaxia (b) and Cx. pallidothorax (c). [file 13071_2019_3625_MOESM3_ESM.pdf]

Figure S1. Predicted secondary structures for 22 tRNAs in *Lt. fuscanus* (A), *Lt. halifaxia* (B) and *Cx. pallidothorax* (C) mt genomes. Nucleotides highlighted in red indicate mismatch.

A

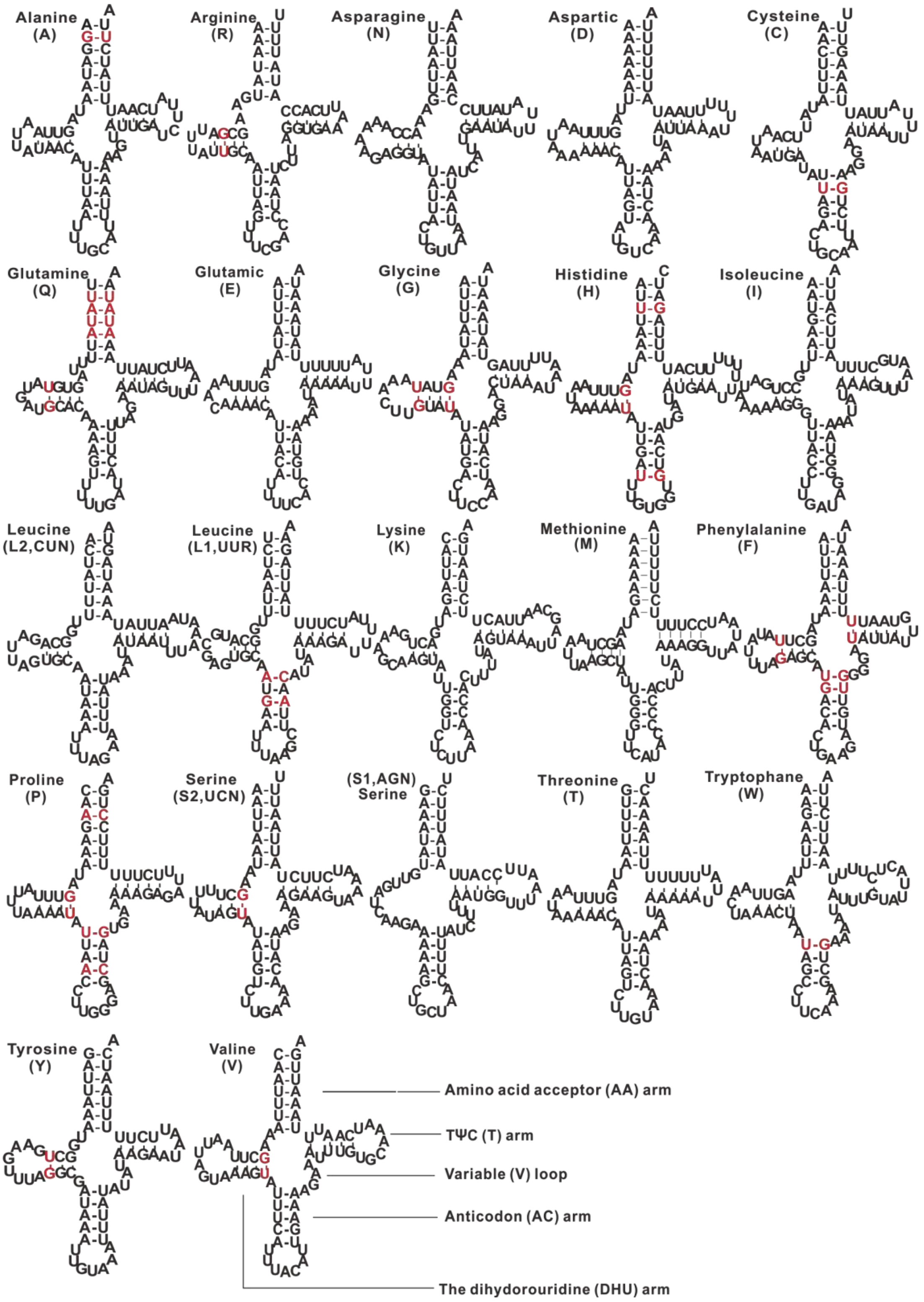

B

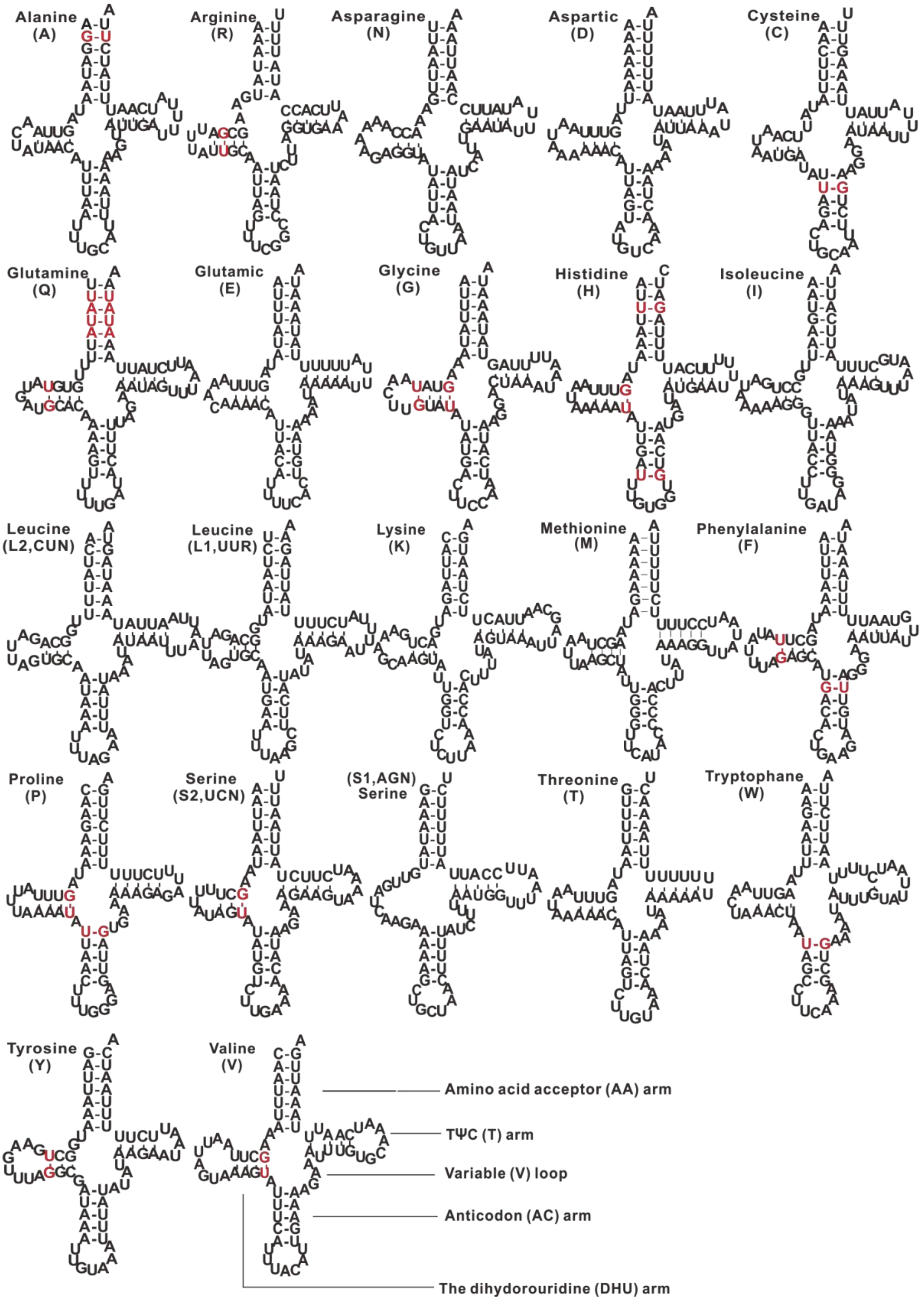

Alanine (A)
Arginine (R)
Asparagine (N)
Aspartic (D)
Cysteine (C)
Glutamine (Q)
Glutamic (E)
Glycine (G)
Histidine (H)
Isoleucine (I)
Leucine (L2,CUN)
Leucine (L1,UUR)
Lysine (K)
Methionine (M)
Phenylalanine (F)
Proline (P)
Serine (S2,UCN)
(S1,AGN) Serine
Threonine (T)
Tryptophane (W)
Tyrosine (Y)
Valine (V)
Amino acid acceptor (AA) arm
TΨC (T) arm
Variable (V) loop
Anticodon (AC) arm
The dihydorouridine (DHU) arm

C

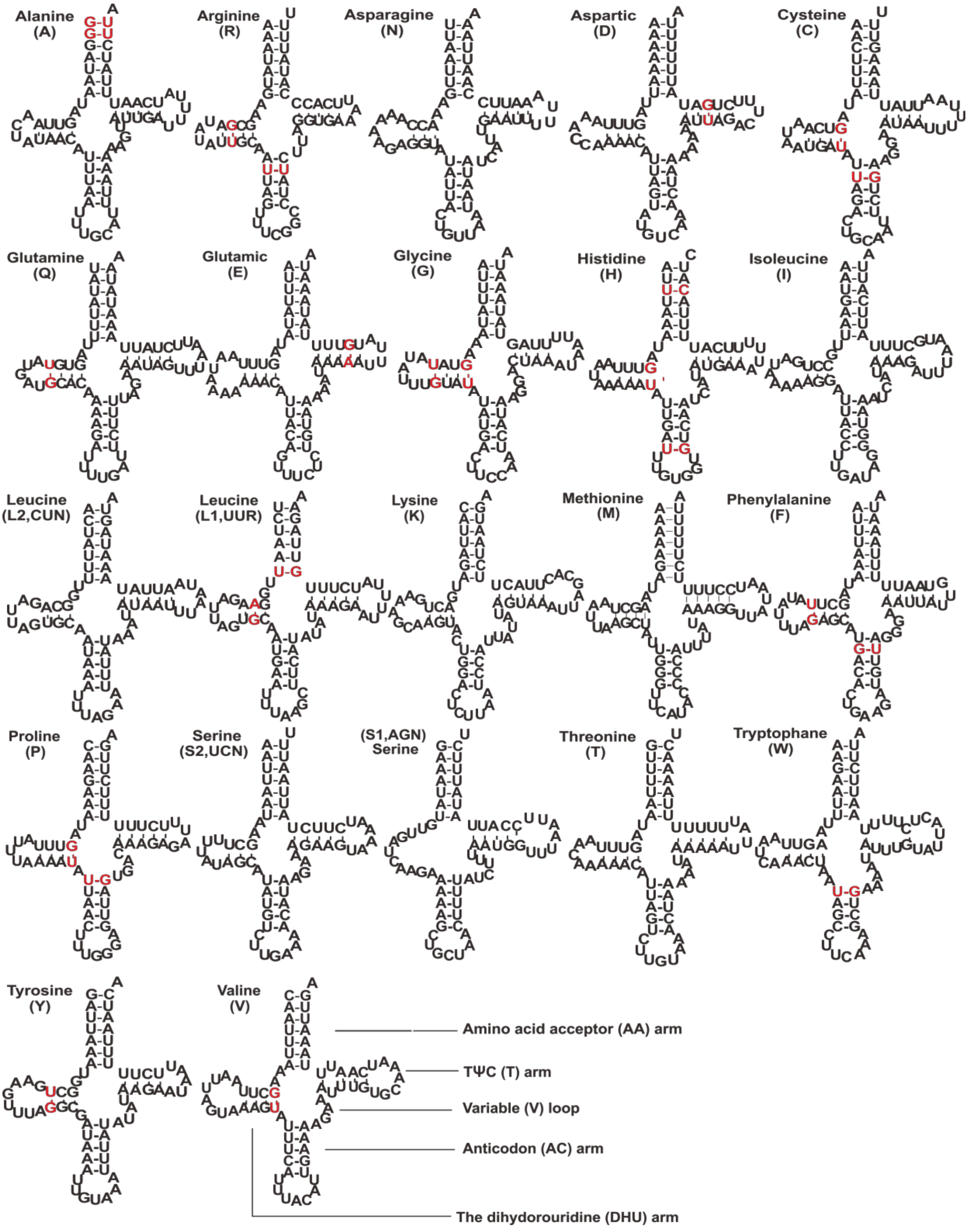
Alanine (A)
Arginine (R)
Asparagine (N)
Aspartic (D)
Cysteine (C)
Glutamine (Q)
Glutamic (E)
Glycine (G)
Histidine (H)
Isoleucine (I)
Leucine (L2,CUN)
Leucine (L1,UUR)
Lysine (K)
Methionine (M)
Phenylalanine (F)
Proline (P)
Serine (S2,UCN)
(S1,AGN) Serine
Threonine (T)
Tryptophane (W)
Tyrosine (Y)
Valine (V)
Amino acid acceptor (AA) arm
TΨC (T) arm
Variable (V) loop
Anticodon (AC) arm
The dihydorouridine (DHU) arm
