# Supplementary material for: The complete mt genomes of Lutzia halifaxia, Lt. fuscanus and Culex pallidothorax (Diptera: Culicidae) and comparative analysis of 16 Culex and Lutzia mt genome sequences
Source: Parasit Vectors. 2019 Jul 26;12:368. doi: 10.1186/s13071-019-3625-2 (PMC6660957; doi:10.1186/s13071-019-3625-2)
Supplement: Supplementary file 4 — Additional file 4: Table S3. Best-fit models chosen under Akaike information criterion by Modeltest for each of the 13 PCGs. [file 13071_2019_3625_MOESM4_ESM.pdf]

**Table S3. Best-fit models chosen under Akaike Information Criterion by Modeltest for each of the 13 PCGs.**

| Gene | Best-fit model |
|---|---|
| *Atp6* | GTR+I |
| *Atp8* | GTR+I |
| *Cox1* | GTR+I+G |
| *Cox2* | TVMef+G |
| *Cox3* | TIM2+G |
| *Cob* | TrN+G |
| *Nad1* | TPM1uf+I |
| *Nad2* | TVM+G |
| *Nad3* | TPM1uf+I |
| *Nad4* | TIM3+G |
| *Nad4l* | F81 |
| *Nad5* | TIM1+I+G |
| *Nad6* | TPM1uf+G |
